# Biopsychosocial Profile of Chronic Alcohol Users: Insights from a Cross-Sectional Study

**DOI:** 10.3390/brainsci15070741

**Published:** 2025-07-10

**Authors:** Luciana Angela Ignat, Raluca Oana Tipa, Alina Roxana Cehan, Vladimir Constantin Bacârea

**Affiliations:** 1 Doctoral School, ‘George Emil Palade’ University of Medicine, Pharmacy, Science and Technology of Targu Mures, 540142 Targu Mures, Romania; 2 ‘Prof. Dr. Alexandru Obregia’ Clinical Psychiatric Hospital, 041914 Bucharest, Romania; 3 Department of Psychiatry, ‘Carol Davila’ University of Medicine and Pharmacy, 020021 Bucharest, Romania; 4 Plastic and Reconstructive Surgery, Emergency Clinical County Hospital of Targu Mures, 540136 Targu Mures, Romania; 5 Department of Scientific Research Methodology, ‘George Emil Palade’ University of Medicine, Pharmacy, Science and Technology of Targu Mures, 540142 Targu Mures, Romania

**Keywords:** alcohol use disorder, relapse, self-efficacy, psychometric tools, readmission prediction

## Abstract

**Introduction:** Chronic alcohol use is a complex condition influenced by psychological, behavioral, and socio-demographic factors. This study aimed to develop a comprehensive psychosocial profile of individuals with alcohol use disorder (AUD) by examining associations between psychometric variables and relapse risk including repeated psychiatric hospitalizations. **Methodology:** A cross-sectional observational analytical study was conducted on a sample of 104 patients admitted for alcohol withdrawal management at the “Prof. Dr. Al. Obregia” Psychiatric Clinical Hospital in Bucharest between March 2023 and September 2024. Participants completed a set of validated psychometric tools: the Drinker Inventory of Consequences—Lifetime Version (DrInC), Readiness to Change Questionnaire—Treatment Version (RTCQ), Drinking Expectancy Questionnaire (DEQ), and Drinking Refusal Self-Efficacy Questionnaire (DRSEQ). Additional data were collected on the socio-demographic (education level, socio-professional category), genetic (family history of alcohol use), and behavioral factors (length of abstinence, tobacco use, co-occurring substance use disorders). **Results:** Higher alcohol-related consequence scores (DrInC) were significantly associated with lower education (*p* < 0.001, η^2^ = 0.483), disadvantaged socio-professional status (*p* < 0.001, η^2^ = 0.514), and family history of alcohol use (*p* < 0.001, η^2^ = 0.226). Self-efficacy (DRSEQ) was significantly lower among individuals with co-occurring substance use (*p* < 0.001) and nicotine dependence (*p* < 0.001). Logistic regression showed that the DrInC scores significantly predicted readmission within three months (OR = 1.09, *p* = 0.001). **Conclusions:** Psychometric tools are effective in identifying individuals at high risk. Personalized, evidence-based interventions tailored to both psychological and socio-professional profiles, combined with structured post-discharge support, are essential for improving long-term recovery and reducing the readmission rates.

## 1. Introduction

Alcohol use disorders (AUDs) remain a critical global public health challenge, with far-reaching consequences on morbidity, mortality, and overall quality of life [1]. According to the World Health Organization (WHO), alcohol consumption was responsible for approximately 2.6 million deaths globally in 2019, representing 4.7% of all deaths. Notably, men were disproportionately affected, accounting for nearly 2 million of these deaths compared with 600,000 among women [1,2].

Globally, it is estimated that over 400 million individuals are currently living with an alcohol disorder. Young adults, particularly those aged 20 to 39, appear to be especially vulnerable; in this age group, alcohol consumption is involved in approximately 13% of all deaths [3].

From a regional perspective, the WHO European Region bears the highest burden of alcohol-attributable mortality, with an annual death rate of 52.9 per 100,000 inhabitants, translating to an estimated 800,000 deaths each year [4]. Per capita alcohol consumption in the European Union stands at 9.2 L annually compared with a global average of 5.5 L. Moreover, in this region, more than one in eleven adults meet the criteria for an AUD [4,5].

AUD is a complex psychiatric condition that not only affects physical health, but also severely disrupts social and psychological functioning, inflicting substantial social and economic costs [6]. Recent research has increasingly studied the broader impact of alcohol use disorder (AUD), especially its effects on quality of life (QoL), encompassing not only medical concerns, but also social and economic consequences [7,8].

Socioeconomic status (SES) has emerged as a fundamental determinant of both alcohol consumption patterns and mental health outcomes, particularly in vulnerable populations [9]. A 2024 study emphasized the complex interplay between SES, alcohol use, and mental health, highlighting that individuals with lower SES backgrounds were more likely to engage in high-risk drinking or abstinence. These behaviors appear to be mediated by factors such as perceived social support and neighborhood conditions [10].

Higher levels of perceived social capital have been associated with fewer AUD symptoms among disadvantaged populations, likely due to their influence on drinking motives, particularly those related to coping and socialization. Conversely, low levels of social capital were linked to increased AUD severity, primarily due to a greater reliance on alcohol as a maladaptive coping strategy [11].

In Romania, a recent study that evaluated the quality of life in hospitalized patients with AUD using the WHOQOL-BREF (World Health Organization Quality-of-Life Scale) instrument noticed moderate scores across the physical, psychological, social, and environmental domains, with significant differences based on marital status. According to these findings, married individuals or those in stable relationships reported a higher quality of life, particularly in the domain of physical health [12].

These findings underscore the complex nature of AUDs and the need for a comprehensive, biopsychosocial approach to treatment that integrates clinical, socio-demographic, and environmental factors. Such an approach is essential for designing effective, personalized interventions to diminish alcohol-related harm, especially among socioeconomically disadvantaged populations [13,14].

In addition to the socio-environmental factors, the neurobiological mechanisms underlying AUDs are essential in understanding relapse [15]. Alterations in brain reward circuitry, heightened impulsivity, and environmental triggers such as alcohol-related cues contribute significantly to relapse risk. Psychological factors, including coping styles, self-efficacy, and social support, further influence the likelihood of relapse, providing important targets for therapeutic interventions [16].

Several studies have highlighted the importance of behavioral change stages in relapse prevention, particularly in the months following discharge from treatment [17]. Patients in the early stages of change, such as contemplation and preparation, are at higher risk of relapse, and those hospitalized for extended periods may develop a dependence on the hospital environment, increasing vulnerability when faced with external stressors post-discharge [18,19].

Moreover, factors like educational background, socio-professional status, family history of alcohol use, and co-occurring dependencies can significantly affect relapse prevention strategies. Expectations regarding alcohol use and self-efficacy in resisting temptation play a critical role in long-term treatment success [20,21].

Modern approaches to relapse view it not as a failure, but as an opportunity to reinforce motivation and adapt treatment plans. Integrating relapse prevention techniques, stress management therapies, and adaptive coping strategies is vital in reducing rehospitalization rates [22].

This study used four validated tools—Drinker Inventory of Consequences (DrInC), Readiness to Change Questionnaire (RTCQ), Drinking Expectancy Questionnaire (DEQ), and Drinking Refusal Self-Efficacy Questionnaire (DRSEQ)—to profile hospitalized individuals with AUDs. Each tool addresses a specific aspect of alcohol use: the long-term consequences of drinking, motivation for change, perceived benefits of alcohol use, and the ability to resist alcohol in high-risk situations. By integrating these tools with clinical and socio-demographic data, this study sought to generate comprehensive insights into the treatment needs of the target population. The findings aim to support the personalization of therapeutic interventions, thereby enhancing individual recovery outcomes and informing more effective institutional resource allocation and planning [17,23,24,25].

An individual’s awareness of alcohol harm (DrInC) and motivation to change (RTCQ) are deeply influenced by their beliefs about alcohol (expectancy) and their confidence to resist it (self-efficacy). High perceived negative consequences and low positive expectancies, combined with strong refusal self-efficacy, are often prerequisites for advancing through the stages of change. Each scale reflects a different psychological domain, but they mutually inform one another: expectancies influence motivation, consequences affect efficacy, and self-efficacy moderates behavioral intentions and outcomes [24,25].

Our study aimed to investigate the complex interrelationships among the consequences of alcohol consumption, readiness for change, drinking expectancies, and self-efficacy in alcohol refusal, while also accounting for relevant socio-demographic and behavioral factors. The overarching objective was to identify reliable predictors of relapse and generate evidence that supports the development of personalized, evidence-based interventions. Such interventions are intended to mitigate relapse risk and promote sustained recovery outcomes in individuals with alcohol use disorders.

## 2. Materials and Methods

This study utilized a cross-sectional, observational design to investigate the associations between sociodemographic and clinical variables and psychological factors related to alcohol use including alcohol-related consequences, expectancies, self-efficacy, and stage of change. In addition, a prospective component was incorporated to identify predictors of rehospitalization within a three-month follow-up period. Sociodemographic variables included education level and socio-professional category, while behavioral and genetic factors encompassed family history of alcohol use, duration of abstinence, co-occurring substance use disorders, and tobacco dependence. The primary objective of this study was to develop a comprehensive clinical and psychosocial profile of individuals with chronic alcohol use who are admitted to psychiatric units for alcohol withdrawal management.

To explore the potential associations between various demographic and clinical variables and the severity of alcohol-related consequences, a series of one-way analyses of variance (ANOVAs) were conducted. The following research hypotheses were formulated for each independent variable:

***H*_1_:**  *There are statistically significant differences in alcohol-related consequence scores across different levels of education.*

***H*_2_:**  *There are statistically significant differences in alcohol-related consequence scores based on socio-occupational category.*

***H*_3_:**  *There are statistically significant differences in alcohol-related consequence scores between individuals with and without a family history of alcohol use.*

***H*_4_:**  *There are statistically significant differences in alcohol-related consequence scores depending on the period of abstinence.*

***H*_5_:**  *There are statistically significant differences in alcohol-related consequence scores based on the presence or absence of co-occurring addictions.*

***H*_6_:**  *There are statistically significant differences in alcohol-related consequence scores between individuals with and without tobacco dependence.*

To investigate how beliefs regarding the effects of alcohol varied across demographic and clinical characteristics, a series of ANOVAs were conducted on alcohol expectancy factors. The aim was to determine whether these expectancy scores significantly differed according to education level, socio-professional category, family history of alcohol use, duration of abstinence, presence of co-occurring substance use disorders, and tobacco dependence. The following hypotheses were formulated to evaluate potential group differences in the alcohol expectancy scores across these variables.

***H*_7_:**  *There are statistically significant differences in specific expectancy factors related to alcohol use across different levels of education.*

***H*_8_:**  *There are statistically significant differences in specific expectancy factors related to alcohol use based on socio-professional category.*

***H*_9_:**  *There are statistically significant differences in specific expectancy factors related to alcohol use between individuals with and without a family history of alcohol use.*

***H*_10_:**  *There are statistically significant differences in specific expectancy factors related to alcohol use depending on the period of abstinence.*

***H*_11_:**  *There are statistically significant differences in specific expectancy factors related to alcohol use based on the presence or absence of co-occurring addictions.*

***H*_12_:**  *There are statistically significant differences in specific expectancy factors related to alcohol use between individuals with and without tobacco dependence.*

Given the central role of self-efficacy in facilitating behavioral change and supporting addiction recovery, this study also explored whether the self-efficacy scores differed significantly across various demographic and clinical groups. A series of ANOVAs were performed to examine differences in self-efficacy based on education level, socio-professional category, family history of alcohol use, duration of abstinence, presence of co-occurring substance use disorders, and tobacco dependence. The following research hypotheses were formulated to assess whether statistically significant differences in self-efficacy existed across these subgroups.

***H*_13_:**  *There are statistically significant differences in self-efficacy scores across different levels of education.*

***H*_14_:**  *There are statistically significant differences in self-efficacy scores based on socio-professional category.*

***H*_15_:**  *There are statistically significant differences in self-efficacy scores between individuals with and without a family history of alcohol use.*

***H*_16_:**  *There are statistically significant differences in self-efficacy scores depending on the period of abstinence.*

***H*_17_:**  *There are statistically significant differences in self-efficacy scores based on the presence or absence of co-occurring addictions.*

***H*_18_:**  *There are statistically significant differences in self-efficacy scores between individuals with and without tobacco dependence.*

To examine potential associations between the individuals’ stage of change and various demographic and clinical characteristics, a series of chi-square tests of independence were conducted. The objective was to assess whether the distribution of participants across the stages of change significantly varied according to education level, socio-professional category, family history of alcohol use, duration of abstinence, presence of co-occurring substance use disorders, and tobacco use status. The following research hypotheses were formulated to evaluate whether the stage of change was significantly associated with each of these variables.

***H*_19_:**  *There is a statistically significant association between stage of change and level of education.*

***H*_20_:**  *There is a statistically significant association between stage of change and socio-professional category.*

***H*_21_:**  *There is a statistically significant association between stage of change and presence of a family history of alcohol use.*

***H*_22_:**  *There is a statistically significant association between stage of change and period of abstinence.*

***H*_23_:**  *There is a statistically significant association between stage of change and presence of co-occurring addictions.*

***H*_24_:**  *There is a statistically significant association between stage of change and presence of tobacco use.*

Rehospitalization among individuals with AUDs remains a significant concern in clinical practice, often reflecting relapses, treatment insufficiency, or inadequate post-discharge support. Identifying the psychological and behavioral predictors of short-term rehospitalization is essential for developing targeted, preventative interventions. Prior research has emphasized the association between alcohol-related consequences, cognitive expectancies related to alcohol use, and self-efficacy on relapse risk and treatment outcomes. Within this framework, the present study aimed to assess whether these psychological factors serve as significant predictors of rehospitalization within three months following discharge. Accordingly, the following predictive hypothesis was formulated.

***H*_25_:**  *Alcohol-related consequences, alcohol expectancy factors, and self-efficacy scores significantly predict the likelihood of rehospitalization within the next three months among individuals with alcohol use disorder.*

### 2.1. Sample and Inclusion/Exclusion Criteria

The study sample included 104 patients admitted to the “Prof. Dr. Al. Obregia” Psychiatric Clinical Hospital in Bucharest between March 2023 and September 2024.

The inclusion criteria were as follows:

• Patients aged 18 years or older;
• A primary admission diagnosis within 24-48 h of hospitalization (according to ICD-10 classification): F10.3—Mental and behavioral disorders due to alcohol use—withdrawal syndrome and/or F10.4—Mental and behavioral disorders due to alcohol use—withdrawal syndrome with delirium;
• Written informed consent to participate in the study.

Exclusion criteria included patients with severe psychiatric comorbidities or cognitive impairments that could interfere with the completion of the assessment tools.

Patients diagnosed with the following ICD-10 codes were excluded: F20—Schizophrenia, F22—Persistent delusional disorders, F23—Acute and transient psychotic disorders, F25—Schizoaffective disorders, F28—Other nonorganic psychotic disorders, F29—Unspecified nonorganic psychosis, F31—Bipolar affective disorder, F32.2—Major depressive episode, severe without psychotic symptoms, F32.3—Major depressive episode, severe with psychotic symptoms, F33.2—Recurrent depressive disorder, current episode severe without psychotic symptoms, F33.3—Recurrent depressive disorder, current episode severe with psychotic symptoms, F70–F73—Intellectual disabilities, and F01–F03—Severe Cognitive Impairments.

The sample size of 104 patients was determined based on the total number of eligible individuals admitted for alcohol withdrawal during the data collection period (March 2023–September 2024) in a ward at the “Prof. Dr. Al. Obregia” Clinical Psychiatric Hospital in Bucharest. A notable limitation of the study is its exclusive focus on male participants, which reflects the demographic characteristics of the psychiatric ward where the research was conducted. This approach reflects the clinical reality and allows the inclusion of all consecutive eligible patients, ensuring a representative sample of chronic alcohol users within the hospital’s population. Furthermore, the sample size was comparable to that used in similar studies from the relevant literature and was considered sufficient for identifying preliminary associations and guiding future research directions. During the data collection period, seventeen patients declined to participate in the study, and five patients were excluded due to severe comorbid psychiatric disorders.

### 2.2. Instruments

The research employed a semi-structured questionnaire specifically designed for this study in combination with four validated psychometric instruments to investigate the socio-demographic characteristics and various dimensions related to AUDs. The validated psychometric instruments used in this study were administered in Romanian, using previously published translations included in the volume “*Mental Health Measurement: A Compendium of Scales and Interviews Used in the Assessment of Psychopathological Disorders* [26]” (Vraști R. www.vrasti.org). The author granted permission for the use of these translated instruments via email on 16 August 2022.

#### 2.2.1. Drinker Inventory of Consequences, Lifetime Version (DrInC)

The DrInC consists of 50 items with binary responses (yes/no) designed to assess the consequences of alcohol consumption across five key areas: intrapersonal, physical, impulse control, social responsibility, and interpersonal (“I have gotten into trouble at work because of my drinking”). Higher scores indicate a greater negative impact of alcohol consumption on an individual’s life. A low score does not necessarily mean that the person has no alcohol-related problems; it may also reflect a lack of awareness or unwillingness to acknowledge the consequences of drinking. This questionnaire was developed as part of Project MATCH, one of the most extensive studies on alcohol treatment, and is used to understand how alcohol affects an individual’s personal, social, and professional life as well as their health. It is a widely recognized and reliable tool for evaluating alcohol-related consequences. Studies have demonstrated its robust internal reliability, with Cronbach’s alpha coefficients ranging from 0.80 to 0.90 across various subscales [27,28,29].

#### 2.2.2. Readiness to Change Questionnaire, Treatment Version (RTCQ)

The RTCQ comprises twelve items with five response options and assesses a patient’s readiness and motivation for behavioral change, particularly concerning reducing or abstaining from alcohol consumption. An example item is: “I am trying to drink less than I used to”*,* rated on a Likert scale from 1 (Strongly Disagree) to 5 (Strongly Agree); the highest subscale score determines the dominant stage. Based on the transtheoretical model of behavior change developed by Prochaska and DiClemente, individuals are categorized into three stages: precontemplation (PC), contemplation (C), and action (A). If the highest score is in the precontemplation stage, the person does not perceive their drinking as problematic and has no intention to change their behavior. A high score in the contemplation stage indicates awareness of the problem but also suggests ambivalence or hesitation about taking immediate action. If the action stage yields the highest score, the individual is actively engaged in the change process and is taking concrete steps toward modifying their drinking behavior [30,31].

#### 2.2.3. Drinking Expectancy Questionnaire (DEQ)

The DEQ consists of 43 items, each with five response options ranging from 1 (Strongly Disagree) to 5 (Strongly Agree). A sample item is: “Drinking helps me feel more outgoing and sociable*”.* This questionnaire evaluates the patient’s expectations and anticipated experiences regarding alcohol consumption across six dimensions: self-confidence, emotional change, increased sexual drive, cognitive change, tension reduction, and dependency. These dimensions can be categorized as either positive or negative, depending on the nature of the expected effects of alcohol consumption. High scores on the positive expectancy dimensions (such as increased self-confidence, cognitive enhancement, and sexual disinhibition) may indicate a predisposition to consume alcohol for its perceived beneficial effects. In contrast, high scores on the negative expectancy dimensions may suggest problematic alcohol use, with alcohol being used as a coping mechanism for stress or indicating a potential tendency toward dependence. The DEQ has demonstrated excellent internal consistency, with Cronbach’s alpha coefficients ranging from 0.78 to 0.93 across its subscales [25,32].

#### 2.2.4. Drinking Refusal Self-Efficacy Questionnaire (DRSEQ)

The DRSEQ contains thirty-one items, each with five response options, assessing a patient’s self-efficacy in refusing alcohol when offered or when it is easily accessible. An illustrative item is: “I could resist drinking when I am at a party where everyone else is drinking”, rated from 1 (Not at all confident) to 5 (Extremely confident), with higher scores indicating greater self-efficacy in refusing alcohol. Conversely, a low score suggests that the individual experiences difficulties in resisting alcohol, particularly in social or emotional contexts. This may reflect a higher risk of problematic alcohol use or relapse following a period of abstinence. The internal consistency of the DRSEQ is high, with Cronbach’s alpha values generally ranging from 0.80 to 0.90, depending on the version and the population studied, indicating very good reliability [33,34].

### 2.3. Monitoring Relapse Risk

To assess the relapse risk, patients were monitored through the hospital’s official electronic system, with follow-up data on the occurrence of rehospitalization recorded within three months post-discharge.

### 2.4. Data Collection Procedure

The questionnaires were administered in a pencil-and-paper format, with no time limits for achievement. These were provided after the resolution of alcohol withdrawal symptoms to ensure accurate and optimal responses. Participants with incomplete responses on key psychometric instruments were excluded from analyses involving those specific variables. The overall rate of missing data was minimal.

### 2.5. Ethical Considerations and Data Protection

The study was approved by the Ethics Committee of the “Prof. Dr. Al. Obregia” Clinical Psychiatric Hospital in Bucharest and followed the ethical and deontological guidelines for medical research. All collected data were coded to maintain confidentiality, in accordance with the General Data Protection Regulation (GDPR).

### 2.6. Statistical Analysis

Data were analyzed using IBM SPSS Statistics software, version 29.0. The significance level was set at *p* < 0.05 for all statistical analyses. The logistic regression model was built in RStudio (version 2024.12.0+467).

Preliminary assumptions: The Kolmogorov–Smirnov test was used to assess the normality of the distribution for continuous variables. As the data met the assumptions for normality, parametric tests were used in subsequent analyses.

Group comparisons: One-way ANOVA was used to test for statistically significant differences in alcohol-related consequences, expectancy, and self-efficacy scores across categorical groups. Effect sizes (η^2^) were calculated to assess the magnitude of differences. When the ANOVA indicated a significant effect, Tukey’s HSD post hoc tests were conducted to identify specific group differences.

Associations: The chi-square test (χ^2^) was used to examine associations between the stage of change and categorical variables. The chi-square analyses met the assumption regarding the expected cell counts, with at least 80% of cells having expected frequencies greater than 5. In cases where this assumption was not met, particularly in 2 × 2 contingency tables, Yates’ continuity correction was applied to adjust the test statistics and provide a more conservative estimate of the association between variables.

Prediction of rehospitalization: Binary logistic regression analysis was used to evaluate whether the alcohol-related consequences, expectancy factors, and self-efficacy scores significantly predicted the likelihood of rehospitalization within 3 months. To enhance the interpretability and statistical validity of the logistic regression model, diagnostic procedures were applied to detect and mitigate multicollinearity. Variance inflation factor (VIF) values were calculated for all predictors. Variables with VIF values exceeding 10 or displaying infinite values were identified as multicollinear and subsequently removed. The alcohol consumption consequence score was retained as a summary index of alcohol-related problems. Expectancy Factor 1—Self-Confidence, which showed statistical or near-statistical significance in the preliminary analyses, was retained in the final model based on its potential clinical relevance.

## 3. Results

The study included 104 participants, with a mean age of approximately 45.8 years. In terms of marital status, the majority of participants identified as either divorced or single, with a lesser number indicating they were married, in a relationship, or widowed. Regarding educational status, a significant segment of participants completed high school or obtained post-secondary qualifications. Most participants were employed, followed by those who were unemployed and retired. It is noteworthy that 17% of individuals were classified as pensioners with disabilities (Table 1).

Concerning substance use, 90% of participants reported that they did not engage in the use of psychoactive substances, while 8% acknowledged the use of marijuana and 2% reported cocaine use. Concerning tobacco consumption, a significant majority of participants identified as smokers (88%), with only a small part having never smoked (7%) or having abstained from smoking for over a year (6%).

In terms of the maximum duration of alcohol abstinence, the participants exhibited a mean abstinence period of 10.8 months. Finally, the study assessed the participants’ intentions regarding a potential return to the hospital within the subsequent three months, with 51 individuals (49%) indicating an affirmative response and 53 individuals (51%) indicating a negative response.

A series of chi-square tests were conducted to explore potential associations between various sociodemographic and clinical variables and hospital readmission within three months (Table 2). There was no statistically significant association between place of origin (rural vs. urban) and hospital readmission (*p* = 0.38). Although the association between marital status and readmission did not reach statistical significance (*p* = 0.068), the result approached the significance threshold, suggesting a potential trend. A statistically significant association was found between education level and hospital readmission (*p* = 0.02), with a contingency coefficient of 0.45, indicating a moderate effect size. The relative risk analysis revealed that patients with a high school education had the highest risk of readmission (relative risk, RR = 1.69). The association between socio-professional status and readmission was not statistically significant (*p* = 0.293). There was no significant association between the use of psychoactive substances (marijuana, cocaine) and hospital readmission (*p* = 0.286). The association between tobacco use and readmission was not statistically significant. A statistically significant association was found between the most extended period of abstinence and hospital readmission (*p* = 0.001). However, the relative risk values were nearly equal across the abstinence categories, suggesting that the effect size was minimal, and the statistical significance may reflect the sample distribution rather than practical differences.

Table 3 provides an overview of the descriptive characteristics of the analyzed variables including the dimensions of self-efficacy, consumption expectancy factors, and the total score for the consequences of alcohol consumption.

Self-efficacy in social pressure situations had a mean score of 34.05, ranging from 20 to 52 and a slightly positively skewed distribution (skewness = 0.62), indicating a tendency toward above-average scores. In contrast, self-efficacy in situations where alcohol consumption was opportunistic had the lowest mean score among the three self-efficacy dimensions (M = 24.33), with a symmetric distribution.

Regarding the consumption expectancy factors, “Self-confidence” recorded the highest mean score (M = 34.29), followed by “Emotional change” (M = 30.84) and “Dependency” (M = 26.33). “Emotional change” also had the highest variability, indicating significant individual differences among participants. Lower scores were recorded for “Increased sexual drive”, “Cognitive change”, and “Tension reduction.”

The analysis revealed that several individual factors were significantly associated with the severity of alcohol-related consequences. Education level showed a statistically significant association with the alcohol consequence scores (*p* < 0.001), with a large effect size (η^2^ = 0.483). Similarly, the socio-professional category was significantly associated with the alcohol consequence scores (*p* < 0.001), yielding a very large effect size (η^2^ = 0.514). Furthermore, the presence of a family history of alcohol consumption (FHAC) was also significantly related to higher alcohol-related consequence scores (*p* < 0.001), with a moderate-to-large effect size (η^2^ = 0.226). Due to the presence of at least one group with fewer than two cases in these variables, post hoc comparisons using Tukey’s HSD could not be conducted.

In contrast, no statistically significant associations were found between alcohol consequence scores and duration of abstinence, presence of other substance use disorders, or tobacco dependence. The statistical details are presented in Table 4.

**Table 4:** Associations between demographic and clinical factors and alcohol consumption consequences.

Variable	*p*-Value	η^2^ (Eta Squared)
Level of education	**<0.001**	0.483
Socio-occupational category	**<0.001**	0.514
Family history of alcohol use	**<0.001**	0.226
Longest period of abstinence	0.593	0.019
Co-occurring dependencies	0.148	0.037
Tobacco dependence	0.472	0.015

Note: Significant *p*-values (*p* < 0.05) are highlighted in bold. The eta-squared coefficient indicates the magnitude of the effect. Interpretation of eta-squared values: small effect (0.01–0.06), moderate effect (0.06–0.14), and large effect (>0.14), according to Cohen 1988 [35] .

Factor 1—Self-confidence: This factor was significantly associated with hereditary and family-related factors (*p* = 0.002), associated dependencies (*p* < 0.001), and nicotine dependence (*p* < 0.001). The effect size was moderate for hereditary and family-related factors (η^2^ = 0.209), while the associations with associated dependencies and nicotine were smaller (η^2^ = 0.157 and η^2^ = 0.134, respectively).

Factor 2—Emotional change: Emotional change was significantly related to hereditary and family-related factors (*p* = 0.040), with a moderate effect size (η^2^ = 0.139). No other predictors showed statistically significant associations with this factor.

Factor 3—Sexual impulse increase: Significant associations were found for education level (*p* < 0.001, η^2^ = 0.207) and socio-professional category (*p* = 0.032, η^2^ = 0.100), suggesting that these variables may play a role in shaping expectations related to increased sexual impulse.

Factor 4—Cognitive change: Cognitive change was significantly associated with hereditary and family-related factors (*p* = 0.002), with a moderate effect size (η^2^ = 0.134).

Factor 5—Tension reduction: Nicotine dependence was significantly associated with tension reduction (*p* = 0.017, η^2^ = 0.078), indicating a small but statistically meaningful relationship.

Factor 6—Dependence: Dependence was associated with education level (*p* = 0.028, η^2^ = 0.103), socio-professional category (*p* = 0.005, η^2^ = 0.186), and, to a lesser extent, other predictors. The complete data are available in Table 5.

**Table 5:** Results of ANOVA analyses for the six expectancy factors related to alcohol use, according to the socio-demographic and clinical variables.

Expectancy Factor	Education Level (p/η^2^)	Socio-Professional Category (p/η^2^)	Family History (p/η^2^)	Abstinence Period (p/η^2^)	Comorbid Dependencies (p/η^2^)	Tobacco Dependence (p/η^2^)
Self-confidence	0.979/0.004	0.105/0.074	**0.002/0.209**	0.128/0.055	**<0.001/0.157**	**<0.001/0.134**
Emotional change	0.206/0.057	0.498/0.033	**0.040/0.139**	0.999/0.000	0.129/0.040	0.470/0.015
Sexual impulse increase	**<0.001/0.207**	**0.032/0.100**	0.074/0.123	0.396/0.029	0.777/0.005	0.117/0.042
Cognitive change	0.169/0.062	0.869/0.012	0.198/0.095	**0.002/0.134**	0.019/0.075	0.078/0.049
Tension reduction	0.728/0.020	0.254/0.052	0.815/0.037	0.105/0.059	0.852/0.003	**0.017/0.078**
Dependence	**0.028/0.103**	0.963/0.006	**0.005/0.186**	0.080/0.065	0.114/0.042	0.083/0.048

Note: Significant *p*-values (*p* < 0.05) are highlighted in bold. The eta-squared coefficient indicates the magnitude of the effect. Interpretation of the eta-squared values: small effect (0.01–0.06), moderate effect (0.06–0.14), and large effect (>0.14), according to Cohen 1988 [35].

• Post Hoc Tests: Alcohol Expectancy and Related Factors

Education level: Tukey HSD post hoc tests were conducted to explore the differences in expectancy factors based on education level. For increased sexual impulsivity, individuals with the lowest level of education reported significantly higher scores compared with all other groups. In contrast, for alcohol dependence expectancy, although the ANOVA revealed a statistically significant overall effect (*p* = 0.028), the post hoc comparisons did not identify any significant pairwise differences between education levels. This suggests that while group-level variation exists, no individual group comparison reached the threshold for statistical significance under the more conservative Tukey test.

Socio-professional status: Tukey HSD post hoc tests examined differences in the expectancy of increased sexual impulsivity across socio-professional categories. Results indicated that retired individuals had significantly lower expectancy scores (M = 10.50) than both daily laborers and unemployed participants, who showed the highest means (M = 14.94 and M = 14.47, respectively). Formally employed individuals and retired individuals with a disability status had intermediate scores (M = 13.24 and M = 13.72), which were not significantly different from either end of the spectrum. These findings suggest that individuals with more stable or institutionalized socio-professional roles may associate alcohol use with less sexual impulsivity than those in more precarious conditions.

Family history of alcohol use: Post hoc comparisons for expectancy factors based on family history of alcohol use (AHC) could not be performed because at least one category had fewer than two participants. This limitation prevented the analysis from meeting the assumptions required for reliable post hoc testing.

Period of abstinence: Tukey HSD tests were used to assess the differences in cognitive change expectancy based on the longest reported period of abstinence. No statistically significant differences were observed, as all abstinence duration groups formed a single homogeneous subset. However, individuals with 1 to 3 years of abstinence reported higher average scores (M = 12.80) compared with those with shorter or longer periods of abstinence (M = 10.19–10.60), suggesting a potential trend that did not reach statistical significance.

Use of psychoactive substances: Post hoc comparisons examined the differences in self-confidence expectancy based on the reported use of psychoactive substances. Participants who reported cocaine use had significantly higher self-confidence scores (M = 47.00) than both non-users (M = 34.05) and cannabis users (M = 33.88), who did not significantly differ from each other. These findings highlight a sharp contrast between stimulant use and other substance categories with respect to perceived self-confidence when drinking alcohol.

Tobacco use: Tukey HSD post hoc analyses explored the differences in self-confidence and cognitive change expectancy according to tobacco use. For self-confidence, current smokers reported significantly higher scores (M = 34.85) than non-smokers (M = 28.75), whereas former smokers (M = 33.00) did not differ significantly from either group. Regarding cognitive change expectancy, no significant differences were found across the smoking status groups. Nevertheless, non-smokers had the lowest average score (M = 8.75) compared with current (M = 10.91) and former smokers (M = 11.60), indicating a possible trend that warrants further investigation.

Self-efficacy in social pressure situations showed no significant association with the analyzed variables. This lack of correlation may indicate the relative autonomy of the perception of social control in the face of familial or socio-professional influences.

Self-efficacy in emotional release situations was significantly associated with socio-professional category (*p* = 0.019), FHAC (*p* < 0.001) and related dependencies (*p* = 0.002), suggesting that individuals from disadvantaged backgrounds or with familial vulnerability perceive a reduced sense of control over consumption behavior in emotionally charged situations. The effects were of small to moderate intensity (η^2^ = 0.111–0.236).

Self-efficacy in situations of alcohol consumption opportunities was significantly correlated with FHAC (*p* = 0.001) and associated dependencies (*p* < 0.001), reinforcing the notion that familial history and poly-substance use negatively affect the ability to resist temptation in contexts favorable to alcohol consumption. The complete data are available in Table 6.

**Table 6:** Socio-demographic and clinical factors associated with self-efficacy in preventing alcohol consumption.

Self-Efficacy Factor	Education Level (p/η^2^)	Socio-Professional Category (p/η^2^)	Family History (p/η^2^)	Abstinence Period (p/η^2^)	Associated Dependencies (p/η^2^)	Tobacco Dependence (p/η^2^)
Social pressure situations	0.172/0.062	0.086/0.078	0.077/0.122	0.796/0.010	0.288/0.024	0.271/0.026
Emotional release situations	0.632/0.025	**0.019/0.111**	**<0.001/0.236**	0.830/0.009	**0.002/0.116**	0.852/0.003
Opportunistic alcohol consumption situations	0.603/0.027	0.185/0.060	**0.001/0.217**	0.278/0.038	**<0.001/0.161**	0.826/0.004

Note. Significant *p*-values (*p* < 0.05) are highlighted in bold. The eta-squared coefficient indicates the magnitude of the effect. Interpretation of the eta-squared values: small effect (0.01–0.06), moderate effect (0.06–0.14), and large effect (>0.14), according to Cohen 1988 [35].

• Post Hoc Tests: Self-Efficacy and Related Factors

Tukey HSD post hoc tests were conducted to examine the differences in emotional release self-efficacy based on socio-professional status. Retired individuals had the lowest mean score (M = 21.00), which was significantly lower than that of all other groups. In contrast, daily laborers reported the highest self-efficacy (M = 33.69), forming a distinct subset. These findings suggest that socio-professional status is associated with perceived emotional competence.

Post hoc comparisons using the Tukey HSD test were conducted to examine differences in self-efficacy in emotional release and drinking-related contexts, based on the use of psychoactive substances. Participants who reported cocaine use consistently exhibited significantly lower self-efficacy scores across both domains—emotional situations (M = 11.00) and alcohol-related situations (M = 12.00)—compared with non-users and cannabis users. Cannabis users showed intermediate levels of self-efficacy, while non-users had the highest scores. These findings suggest a potential negative association between substance use, particularly stimulant use, and perceived emotional and behavioral control.

The chi-square test was used to analyze the relationship between the stages of change in alcohol consumers and various socio-demographic and clinical variables. The results are summarized in Table 7.

Among the analyzed variables, only rehospitalization within the first 3 months after discharge was found to have a statistically significant association with the stages of change (*p* < 0.001) with the highest contingency coefficient (C = 0.714), indicating a strong relationship. This finding suggests that the risk of early relapse is closely linked to the stage at which an individual is in the behavioral change process related to alcohol consumption.

The ANOVA results indicated that the number of hospitalizations since October 2021 had a statistically significant effect on the alcohol-related consequence scores (*p* = 0.001), with moderate explanatory power (R^2^ = 0.354, adjusted R^2^ = 0.226). This finding suggests that variations in the number of hospitalizations are meaningfully associated with differences in the perceived consequences of alcohol use. However, due to the presence of groups with fewer than two cases, post hoc comparisons could not be conducted, limiting the ability to interpret specific group differences.

Additionally, a significant difference was observed between individuals who were rehospitalized within three months and those who were not (*p* < 0.001). This binary variable was significantly associated with the alcohol consequence scores, although the explanatory power of the model was modest (R^2^ = 0.144, adjusted R^2^ = 0.135). These findings suggest that short-term rehospitalization is related to differing perceptions of alcohol-related consequences, potentially reflecting variations in the severity or awareness of alcohol-related harms among these groups. The complete data are available in Table 8.

**Table 8:** Analysis of variance of consequence scores based on number of hospitalizations and readmission status.

Independent Variable	p (Sig.)	η^2^ (R Squared)	Notes
Number of hospitalizations since October 2021	**0.001**	0.354 (adjusted: 0.226)	Some groups had fewer than 2 cases; therefore, post-hoc tests were not conducted.
Readmission within the next 3 months (Yes vs. No)	**<0.001**	0.144 (adjusted: 0.135)	Significant difference observed based on readmission status.

Note: η^2^ (eta-squared) represents the effect size, with 0.01–0.06 = small, 0.06–0.14 = moderate, and >0.14 = large effects (Cohen, 1988 [35]). The adjusted R^2^ values reflect the proportion of variance in the consequence score explained by the independent variables. Post hoc comparisons were not conducted for hospitalization frequency due to small group sizes (<2 cases per group). Bold values indicate statistically significant results (*p* < 0.05).

A binary logistic regression analysis was applied to examine the relationship between the psychological variables and the likelihood of readmission. Readmission was treated as a dichotomous dependent variable (0 = no, 1 = yes), and the scores obtained from the psychological scales were introduced as independent variables. The logistic regression model identified the alcohol consumption consequence score as a statistically significant predictor of psychiatric readmission within three months (OR = 1.09, *p* = 0.001, 95% CI: 1.03–1.15). Each additional point on this scale increased the odds of readmission by approximately 9%. While the self-confidence expectancies showed a positive association with readmission (OR = 1.09), this effect did not reach statistical significance (*p* = 0.093), suggesting a potential but inconclusive relationship. The complete data are available in Table 9.

**Table 9:** Logistic regression results predicting the likelihood of readmission within 3 months.

	Coefficient B	Standard Error	*p*-Value	Odds Ratio	95% Conf. Interval
Constant	−5.07	1.76	0.004	0.01	0–0.2
Alcohol Consumption Consequence Score	0.09	0.03	**0.001**	1.09	1.03–1.15
Alcohol Expectancy Factor 1 (Self-Confidence)	0.09	0.05	0.093	1.09	0.99–1.2

Notes: Coefficient—The value of the regression coefficient for each variable. Odds ratio (OR)—The odds ratio represents the likelihood of the outcome event occurring with a one-unit increase in the predictor. Bold values indicate statistically significant results (*p* < 0.05).

## 4. Discussion

This study aimed to explore the complex interrelationships among the psychological, behavioral and sociodemographic factors influencing alcohol-related outcomes in individuals admitted for inpatient alcohol withdrawal treatment. Using a cross-sectional observational design with a prospective component, the analysis focused on variables such as alcohol-related consequences, drinking expectancies, self-efficacy in alcohol refusal, and stage of change, while also considering their association with relapse and rehospitalization within three months post-discharge. The ultimate goal was to develop a comprehensive psychosocial profile to inform personalized, evidence-based interventions that enhance long-term recovery.

### 4.1. Alcohol-Related Consequence Scores

Education level, socio-professional category, and FHAC showed significant differences in alcohol-related consequence scores, as indicated by the ANOVA results. Education level alone accounted for nearly 48% of the variance in the alcohol consequence scores, highlighting the substantial role that cognitive, informational, and social resources linked to educational status may play in shaping the individuals’ vulnerability to alcohol-related harm. Likewise, the socio-professional category demonstrated an even more substantial effect, underscoring the importance of occupational roles and associated lifestyle factors such as job stress, economic stability, and access to health and social support systems.

The presence of FHAC was also significantly associated with more severe alcohol-related consequences, suggesting that intergenerational behavioral patterns or genetic predispositions may contribute to heightened risk. In contrast, other clinical or behavioral variables, including duration of abstinence, tobacco dependence, and co-occurring substance use disorders, did not show statistically significant associations with the consequence scores and yielded only minimal effect sizes.

These findings align with broader trends reported in the literature concerning the disproportionate burden of alcohol-related harm experienced by socioeconomically disadvantaged populations. Individuals with lower levels of educational status who engage in harmful alcohol use are exposed to elevated health risks that exceeded the anticipated additive effects of low education and alcohol consumption independently. This compounded vulnerability persisted, even after adjusting for relevant health behaviors and additional socioeconomic covariates, indicating a robust interaction between social disadvantage and alcohol-related harm. Furthermore, a steeper risk gradient was observed among participants with lower educational accomplishment, suggesting that these individuals may possess increased biological or social susceptibility to the adverse effects of alcohol [36,37].

Moreover, although individuals from higher socioeconomic backgrounds tend to consume alcohol more frequently, those from lower socioeconomic status (SES) groups experience disproportionately greater health-related harm, even at similar or lower levels of consumption. For instance, heavy drinkers residing in socioeconomically deprived areas exhibit significantly higher rates of alcohol-related hospitalizations and mortality compared with their more affluent counterparts. This phenomenon, commonly referred to as the alcohol harm paradox, underscores the role of social context in exacerbating the health consequences of alcohol use, beyond the influence of consumption patterns alone [38,39].

At a structural level, national-level socioeconomic disparities also appear to be associated with alcohol-related outcomes. Countries characterized by higher levels of income inequality tend to report greater prevalence of harmful alcohol use, with the relationship between low SES and risky drinking behaviors being more pronounced in societies with elevated social disparities. These findings highlight the importance of macro-level determinants, including social cohesion, equitable access to healthcare, and supportive policy infrastructure, in mitigating alcohol-related harm and reducing the health burden among disadvantaged populations [40].

A family history of problematic alcohol use emerged as a significant factor associated with more severe alcohol-related consequences, a finding that is well-supported in the literature. Research dating back to the 1990s has consistently demonstrated that children of individuals with alcohol use disorders are at markedly higher risk for a range of psychological and behavioral difficulties compared with their peers. These individuals are more likely to engage in the early initiation of alcohol use, exhibit higher frequencies of drinking, and display elevated rates of substance use and abuse. In addition, they are more prone to emotional and behavioral disorders including depression, anxiety, and antisocial behaviors [41,42].

Both genetic predisposition and environmental influences have been identified as key contributors to these elevated risks. Exposure to parental drinking, family dysfunction, and interpersonal conflict during the formative years further exacerbates the vulnerability to harmful alcohol-related outcomes [42]. Supporting the role of heritability, research has shown that genetic factors account for a substantial proportion of the variance in alcohol dependence, particularly in more severe presentations of the disorder. However, shared environmental factors, such as familial norms and parenting styles, also play a critical role, particularly in the initiation and early patterns of alcohol use [43].

The Whitehall II study provided compelling evidence for the detrimental impact of combined substance use on cognitive functioning in older adults. Specifically, individuals who engaged in both heavy alcohol consumption and smoking exhibited a 36% faster rate of cognitive decline compared with those who abstained from both behaviors [44]. These findings highlight the synergistic effects of alcohol and tobacco use on long-term brain health. Further research has consistently shown that alcohol dependence and smoking are each independently associated with increased all-cause mortality. When combined, these behaviors significantly elevate the risk of premature death, underscoring their additive and possibly multiplicative harm [45]. In individuals with alcohol dependence, such cognitive impairments are particularly concerning, as they can compromise executive functions, including planning, attention, and memory, which are critical for maintaining recovery and engaging in treatment [46].

Despite the strong evidence linking smoking and alcohol use to adverse health outcomes, the present study did not identify a significant association between tobacco dependence and the severity of alcohol-related consequences. This finding suggests that while smoking is a prevalent comorbidity among individuals with alcohol use disorders, its direct association with the acute consequences of alcohol consumption may be limited or mediated by other variables. Nevertheless, the broader health risks associated with concurrent tobacco and alcohol use warrant continued attention in both clinical assessment and intervention strategies.

Tobacco use may indirectly influence the alcohol outcomes by exacerbating cognitive impairments or emotional dysregulation, but may not necessarily contribute directly to alcohol-related consequences such as legal problems, social dysfunction, or injury. In this context, tobacco’s role may be better understood as a comorbid risk factor rather than as a direct moderator of alcohol-related harm [47,48].

Additionally, some evidence suggests that psychosocial variables, such as stress, trauma history, or psychiatric comorbidity, may mediate the relationship between tobacco and alcohol use [49]. Although tobacco use is closely intertwined with alcohol use in many populations, its lack of association with alcohol-related consequences in this study may reflect a combination of measurement focus, sample characteristics, and mediating variables that warrant further investigation in longitudinal or multivariate models.

These findings underscore the importance of adopting a biopsychosocial model when assessing alcohol-related harm. Interventions focused solely on individual behavior may fall short unless they are complemented by socially responsive strategies that address educational and occupational disparities. A deeper understanding of how the sociodemographic context interacts with psychological readiness for change, expectancy beliefs, and refusal self-efficacy is crucial for designing personalized, equitable interventions aimed at preventing relapse and promoting sustainable recovery.

### 4.2. Alcohol Expectancies

In the present study, the “Increased Sexual Impulse” factor from the DEQ was found to be significantly associated with both the educational level and socio-professional category. These associations suggest that educational and occupational status may be related to differences in impulse regulation and alcohol-related expectancies, particularly in contexts involving sexual arousal or behavior. Individuals with higher levels of education or more stable socio-professional positions may exhibit greater awareness, cognitive control and self-regulatory capacity, thereby moderating impulsive behaviors including those linked to alcohol use. In contrast, lower educational status or occupational instability may be associated with more disinhibited or disorganized patterns of alcohol use, potentially driven by stronger or less regulated sexual and emotional impulses.

These findings are consistent with previous research indicating that impulsivity, educational background, and SES significantly shape substance use behaviors. Traits such as sensation seeking and lack of premeditation, key dimensions of impulsivity, have been robustly linked to problematic alcohol use, particularly in individuals with strong sex-related alcohol expectancies [50]. Furthermore, educational status has been shown to serve as both a risk and protective factor. At the same time, higher education is often correlated with greater overall alcohol consumption; it is also associated with enhanced self-regulation and a lower risk of alcohol-related harm [51]. Additionally, early-life socioeconomic indicators, such as lower parental education and household income, have been associated with the earlier initiation of alcohol use, smoking, and sexual activity during adolescence, reflecting the long-term influence of socio-educational context on behavioral risk profiles [52,53,54].

A nuanced understanding of how factors such as impulsivity, sexual expectancies, and social disadvantage interact can guide the development of targeted interventions aimed at reducing risk among vulnerable populations.

The findings from this study also suggest that individuals who have maintained more extended periods of abstinence (1–3 years) are more likely to report pronounced cognitive effects associated with prior alcohol use, particularly when compared with those who have abstained for shorter durations (1–6 months). This may reflect a heightened critical awareness of alcohol’s effects on cognitive domains such as memory, mental clarity, and concentration among long-term abstainers. As individuals progress in their recovery, the contrast between their cognitive functioning during abstinence and their prior state during active use may become more salient, reinforcing their motivation to maintain sobriety.

Empirical evidence supports the notion that long-term abstinence contributes to cognitive recovery. Improvements have been documented in areas such as attention, executive functioning, and working memory, although some deficits, particularly in spatial processing and visuospatial tasks, may persist despite extended periods of sobriety [55]. Furthermore, studies indicate that the expectation of alcohol’s positive cognitive effects diminishes as abstinence is maintained, suggesting that prolonged sobriety may alter both the perceived benefits and risks associated with alcohol consumption [56,57]. These findings highlight the role of cognitive insight and shifting alcohol expectancies as potential mechanisms for sustaining abstinence over time.

### 4.3. Drinking Refusal Self-Efficacy

Drinking refusal self-efficacy has been consistently recognized in the literature as a key psychological factor in the prevention and management of alcohol use disorders. The current study contributes to this knowledge by highlighting the context-dependent nature of self-efficacy in alcohol-related behaviors and its associations with socio-demographic and behavioral variables. In our study, self-efficacy in social pressure situations did not demonstrate a significant association with any of the analyzed variables including FHAC and socio-professional status. This absence of correlation may indicate a relative autonomy of social resistance skills from socio-demographic influences.

These findings align with those of Chen et al., who found that alcohol resistance self-efficacy moderated the relationship between perceived peer drinking norms and binge drinking. Specifically, individuals with higher self-efficacy in social contexts were less affected by peer behaviors, suggesting that strong self-regulatory capacity can serve as a protective buffer against external pressures, regardless of social or familial background [58].

In contrast, self-efficacy in emotionally stressful situations was significantly associated with socio-professional status, FHAC, and co-occurring substance use disorders, according to our results. Participants from disadvantaged socio-professional categories or with familial vulnerability reported lower emotional self-efficacy, suggesting that these factors may compound difficulties in emotional regulation, a well-established risk factor in the initiation and escalation of alcohol use. Post hoc analyses further revealed that retired individuals had the lowest emotional self-efficacy, while daily laborers reported the highest. This pattern may be associated with differences in daily structure, occupational demands, and levels of social engagement, which could be related to variations in the individuals’ perceived emotional competence.

Previously, it was demonstrated that both positive alcohol expectancies and lower self-efficacy independently predicted the onset and escalation of alcohol misuse in young adolescents, highlighting the relevance of these psychological constructs as early risk indicators [59,60]. In light of these findings, the present study supports the clinical value of integrating expectancy and self-efficacy measures into early screening protocols.

Additionally, self-efficacy in opportunistic drinking situations where alcohol is readily available or socially encouraged was significantly associated with FHAC and co-occurring substance use, reflecting a pattern in which familial predisposition and poly-substance use undermine the ability to resist alcohol in high-risk environments. Post hoc analyses showed that participants with a history of cocaine use exhibited the lowest self-efficacy scores across both emotional and opportunity-related domains compared with cannabis users and non-users. These individuals may be especially vulnerable to using substances as a form of emotion regulation, particularly to avoid negative affect or emotional discomfort.

The broader literature supports this association between self-perception and substance use. For example, it has been reported that beliefs about others’ success in recovery (other-efficacy) were more predictive of post-treatment alcohol use than self-efficacy alone, emphasizing the influence of social context and interpersonal expectations on recovery outcomes [61,62]. Additional studies have demonstrated that low self-esteem is associated with higher alcohol consumption, particularly among young adults, and that such patterns may vary across genders [63]. Furthermore, low self-esteem has been shown to mediate the relationship between loneliness and problematic alcohol use, reinforcing the importance of targeting self-concept and emotional regulation in therapeutic interventions [64,65].

Taken together, these findings underscore the importance of context-specific assessments of self-efficacy in both clinical evaluation and treatment planning. Tailoring interventions to strengthen self-regulation in emotionally and situationally vulnerable contexts, especially among individuals with familial or socio-occupational risk factors, may significantly enhance the treatment efficacy and reduce the relapse rates. Future research and clinical practice would benefit from integrating self-efficacy profiles into individualized care strategies, thereby improving the recovery outcomes and long-term resilience.

### 4.4. Stages of Change and Early Relapse Risk

The findings of the present study indicate that relapse within the first three months following discharge is significantly associated with individuals being in the earlier stages of behavioral change such as contemplation or preparation. This supports the theoretical framework of the transtheoretical model (TTM) proposed by Prochaska and DiClemente, which identifies an individual’s stage of change as a critical determinant of treatment outcomes and relapse vulnerability. Patients in pre-action stages, who have not yet committed to or initiated behavioral change, are particularly susceptible to early relapse during the high-risk period following inpatient detoxification [17].

Recent empirical studies further underscore the importance of both psychological readiness and clinical indicators in predicting the post-treatment outcomes for individuals with alcohol use disorders. These variables have been shown to play a substantial role in determining relapse trajectories, emphasizing the need for continued support and monitoring in the immediate post-discharge period, particularly for individuals in the early stages of change [18,19]. This complexity necessitates a more personalized approach to relapse prevention and long-term recovery planning.

Of particular note is the work of Witkiewitz and colleagues, who have advanced the concept of non-abstinent recovery, proposing that clinically meaningful improvements can occur even in the absence of complete abstinence. This perspective challenges traditional binary definitions of success in addiction treatment. It aligns with earlier findings that support gradual reductions in alcohol consumption as a viable and beneficial recovery pathway for specific individuals [20,21]. These insights have significant implications for clinical practice, suggesting that recovery interventions should be tailored to the individual’s stage of recovery that are flexible and responsive to their unique goals and contexts.

### 4.5. Predictors of Readmission and Clinical Utility of Consequence Scores

By systematically addressing multicollinearity, the refined logistic regression model achieved greater statistical stability in coefficient estimation, an enhanced interpretability of individual predictor effects, and adhered to best practices in psychological and medical research. These improvements enhance the model’s clinical utility, especially in identifying individuals at elevated risk for psychiatric readmission based on psychological and behavioral characteristics.

The alcohol consumption consequence score emerged as a robust, independent predictor of psychiatric readmission within three months, highlighting the significant role of cumulative alcohol-related consequences in short-term relapse and hospitalization risk. Although Expectancy Factor 1—Self-Confidence did not meet the conventional threshold for statistical significance (*p* < 0.05), its positive coefficient suggests a potential psychological mechanism influencing readmission risk. This trend warrants further investigation in larger samples and with extended models that incorporate additional psychological predictors.

Our analysis found that the number of prior hospitalizations and readmissions within three months significantly correlated with higher scores on the DrInC scale. This aligns with existing studies indicating that individuals with AUDs face a heightened risk of readmission, functional impairment, and psychosocial instability [66]. Frequent hospitalizations may signal underlying severity, lack of social support, or comorbid psychiatric conditions. Furthermore, readmission within a short window post-discharge appears to be a critical indicator of early vulnerability, underscoring the need for structured follow-up programs to support sustained abstinence and prevent relapse.

The alcohol consequence score not only reflects the clinical status, but also the broader psychosocial correlates of drinking, making it a valuable tool for predicting patterns of psychiatric service use. Several studies have emphasized that systemic shortcomings, such as fragmented care pathways and insufficient post-discharge support, contribute significantly to elevated readmission rates among individuals with substance use or mental health disorders. Conversely, the implementation of coordinated aftercare strategies, particularly those involving community-based mental health services, has been shown to substantially reduce the risk of rehospitalization. These findings underscore the importance of continuity of care and integrated service delivery in supporting long-term recovery outcomes [67,68].

Interestingly, the present findings revealed a paradoxical association between higher levels of self-efficacy, particularly in emotionally and socially challenging situations, and an increased likelihood of readmission during the follow-up period. This counterintuitive result challenges the conventional assumption that higher self-efficacy is universally protective in addiction recovery.

One possible explanation lies in the concept of overconfidence, whereby individuals with an inflated sense of control over their sobriety may underestimate their vulnerability to relapse, leading to reduced adherence to treatment, premature disengagement from support systems, or exposure to high-risk situations without adequate coping strategies. This phenomenon, sometimes referred to as illusory control or false confidence, has been observed in various behavioral domains, including substance use, and may mask underlying vulnerabilities or emotional dysregulation [68]

Additionally, self-reported self-efficacy may not always reflect the actual behavioral competence, particularly in the early stages of recovery. Elevated scores may result from denial, social desirability bias, or limited insight into one’s triggers and limitations. In such cases, high self-efficacy may be more indicative of risk than resilience, particularly when it is not supported by sustained behavioral change or engagement in relapse prevention strategies [69,70]. Supporting this interpretation, prior research has shown that individuals with lower initial self-efficacy often display greater treatment engagement, possibly due to heightened motivation and a greater reliance on structured therapeutic support. This suggests that recognizing personal limitations can, paradoxically, lead to better recovery outcomes [71].

Clinically, these findings underscore the importance of evaluating not only the level of self-efficacy, but also its validity and accuracy. Overconfidence should be viewed as a potential relapse risk factor, and interventions should aim to develop a calibrated, self-efficacy, realistic, experience-informed sense of confidence that aligns with the actual coping skills. Techniques such as motivational interviewing, cognitive-behavioral therapy (CBT), and structured relapse prevention programs can help individuals build a more accurate self-appraisal and develop adaptive coping strategies. Moreover, routine follow-up assessments should monitor discrepancies between perceived and demonstrated self-regulation, enabling early intervention where overestimated self-efficacy may threaten the recovery outcomes [69,72].

### 4.6. Integrated Interpretation and Clinical Application

By integrating ANOVA and logistic regression analyses, the present study captured both the descriptive group differences and predictive patterns related to relapse risk. The consistent observation that alcohol-related consequence scores and self-efficacy perceptions significantly predicted readmission within the follow-up period underscores the clinical relevance of these psychological constructs in treatment and aftercare planning.

These findings suggest that effective interventions should extend beyond addressing substance use behaviors alone. Specifically, they should also target the individuals’ cognitive appraisals of alcohol-related consequences and their self-regulatory beliefs, which appear to play a critical role in determining recovery trajectories. Special attention should be given to patients who demonstrate a tendency to overestimate their coping abilities, as inflated self-efficacy may hinder engagement with treatment or increase exposure to high-risk situations. Integrating these psychological assessments into routine clinical evaluations may enhance the identification of high-risk individuals and support the development of personalized, cognitively informed relapse prevention strategies.

## 5. Study Limitations

Despite the relevance and significance of the findings, several limitations must be considered when interpreting the results of this study. First, the cross-sectional design limits the ability to infer causal relationships among the variables under investigation. While associations can be identified, it cannot be conclusively determined whether psychological or socio-professional factors are related to or contribute to differences in alcohol consumption behaviors or relapse risk. Second, the use of self-report instruments to assess psychological and socio-professional characteristics introduces the potential for response bias, as participants may answer in ways that align with social desirability or self-presentation motives, which could compromise the objectivity of the data.

Another notable limitation is that the study was conducted exclusively with male participants, reflecting the demographic profile of the psychiatric ward where the research was carried out. As such, the findings may not be generalizable to female populations, particularly given the well-documented sex and gender differences in alcohol use patterns, clinical manifestations, and treatment responses. While our findings provide important insights into a male clinical population, we acknowledge the need for future research that includes gender-diverse samples to facilitate comparisons and more representative conclusions. In addition, the sample lacked socio-professional diversity, with certain groups, such as retirees, being underrepresented. This imbalance may have distorted the estimates of group differences and limited the applicability of the results to broader population subgroups.

Another limitation of this study was the relatively small sample size, which reduced the statistical power and restricted the ability to conduct advanced psychometric analyses such as measurement invariance testing. Finally, the absence of a priori power analysis represents a methodological limitation, as it prevented the evaluation of whether the sample size was adequate to detect meaningful effects with sufficient statistical power. Addressing these limitations in future research would improve the validity, reliability, and generalizability of the findings.

## 6. Future Research Directions

To extend and validate the findings, future research should focus on longitudinal studies that track the evolution of patients over time, allowing for the testing of causal relationships between psychological factors and consumption behaviors. Such studies would help clarify the role of psychological variables in predicting relapse and support the development of more appropriately individualized therapeutic interventions.

It would also be beneficial to include a larger and more socio-culturally diverse sample in order to assess variations in the psychological and behavioral factors across different population groups. Another important research direction would be to explore the interaction between the psychological variables and coping mechanisms that patients use to manage stress and social pressure, given that these mechanisms can be closely associated with the progression of consumption behavior.

The clinical usefulness of the DrInC as a predictive tool for readmission risk is both pertinent and promising. Given its ability to capture the multidimensional consequences of alcohol use, including the physical, interpersonal, intrapersonal, social responsibility, and impulse control domains, the DrInC offers a comprehensive psychosocial profile that can inform both risk stratification and treatment planning. In the context of this study, higher DrInC scores were associated with more severe alcohol-related consequences and may reflect an elevated risk of early relapse or rehospitalization. To capitalize on this predictive value, the DrInC could be formally integrated into discharge planning protocols, serving as a standardized measure for identifying individuals who require enhanced post-discharge monitoring or intensive outpatient follow-up.

Clinically, incorporating the DrInC into routine assessment at intake and before discharge could help practitioners adapt individualized care plans, particularly for those with high scores in domains like impulse control or intrapersonal distress, areas often linked to relapse. Furthermore, serial administration of the DrInC during early recovery follow-ups would enable clinicians to monitor changes in the severity of consequences over time, facilitating the early detection of deterioration and timely intervention. Integration into electronic health records could also enable automated alerts when scores exceed a clinically defined threshold, prompting additional support such as brief interventions, case management, or motivational interviewing. Future research could explore the development of cut-off scores or risk indices derived from the DrInC to further improve its utility as a proactive relapse prevention tool.

## 7. Conclusions

In conclusion, the findings of this study highlight the critical importance of integrating psychological and socio-professional variables into the clinical assessment and management of individuals with problematic alcohol use. The psychometric instruments employed, notably those measuring self-efficacy and the perceived consequences of alcohol consumption, demonstrated strong potential for identifying individuals at heightened risk of relapse and rehospitalization.

These results reinforce the clinical utility of such tools not only for the early identification of high-risk profiles, but also for the formulation of personalized therapeutic interventions. By systematically incorporating these measures into both initial evaluations and post-discharge monitoring protocols, clinicians can adjust treatment strategies to the unique psychological and contextual needs of each patient.

Ultimately, this study supports the adoption of a multidisciplinary and individualized approach to care, one that integrates medical, psychological, and social domains, as essential to optimizing the recovery outcomes and minimizing the risk of recurrent hospitalizations in this population.

## Figures and Tables

**Table 1:** Socio-demographic profile and behavioral characteristics of participants.

Variable	Category	Frequency	Percentage
Age	26–30	2	1.9%
	31–40	25	23.1%
	41–50	50	46.3%
	51–60	22	20.4%
	over 60	5	4.6%
Residence area	Rural	37	35.2%
	Urban	67	64.8%
Marital status	Married	22	20.4%
	In a relationship	6	5.6%
	Separated	4	3.7%
	Divorced	35	32.4%
	Single	36	33.3%
	Widowed	1	0.9%
Educational level	1–4 grades	4	3.7%
	1–8 grades	25	23.1%
	High school	33	30.6%
	Post-secondary	25	23.1%
	University degree	17	15.7%
Socio-occupational status	Employed (official contract)	51	47.2%
	Unemployed	17	15.7%
	Retired	2	1.9%
	Retired with disability	18	16.7%
	Day laborer	16	14.8%
Psychoactive substance use	Cocaine	2	1.9%
	Marijuana	8	7.4%
	Denies using other substances	94	87.0%
Tobacco use	Smoker	91	84.3%
	Never smoked	7	6.5%
	Abstinent for over 1 year	6	5.6%
Longest abstinence period	1–6 months	58	53.7%
	7–12 months	21	19.4%
	1–3 years	20	18.5%
	Over 3 years	5	4.6%
Readmission within 3 months	YES	51	47.2%
	NO	53	49.1%

**Table 2:** Chi-square test results examining associations between sociodemographic and clinical variables and psychiatric readmission within three months.

Variable	χ^2^	df	*p*-Value	Contingency Coefficient (C)
Place of origin	0.77	1	0.38	0.12
Marital status	7.12	3	0.068	0.36
Education level	11.62	4	**0.02**	0.45
Socio-professional category	4.95	4	0.293	0.30
Psychoactive substance use	2.51	2	0.286	0.22
Tobacco use	4.48	2	0.107	0.29
Abstinence duration	17.7	4	**0.001**	0.54
Stage of change	4.02	2	0.134	0.27

Note: Significant *p*-values (*p* < 0.05) are highlighted in bold.

**Table 3:** Descriptive statistics for psychosocial constructs related to alcohol use: self-efficacy, expectancy dimensions, and drinking consequences.

Psychosocial Variables	N	Min	Max	M	SD	Skewness	Kurtosis
**Self-efficacy dimensions according to the DRSEQ:**							
Self-Efficacy in Social Pressure Situations	104	20	52	34.05	6.156	0.618	0.611
Self-Efficacy in Emotional Release Situations	104	11	50	28.75	7.914	0.298	0.157
Self-Efficacy in Opportunistic Alcohol Consumption Situations	104	12	35	24.33	4.449	−0.008	0.377
**Factors of expectancy according to the DEQ:**							
Factor 1—Self-Confidence	104	24	47	34.29	4.519	0.519	10.346
Factor 2—Emotional Change	104	16	54	30.84	8.403	0.282	−0.311
Factor 3—Sexual Impulse Increase	104	5	19	13.73	2.528	−0.620	0.826
Factor 4—Cognitive Change	104	4	20	10.78	2.734	0.618	10.448
Factor 5—Tension Reduction	104	7	16	11.23	2.091	0.401	0.019
Factor 6—Dependence	104	20	32	26.33	2.967	−0.262	−0.147
**Alcohol consumption consequences score according to the DrInC:**	104	3	39	24.17	9.031	−0.439	−0.564

Note: N = number of participants; Min = minimum score; Max = maximum score; M = mean; SD = standard deviation.

**Table 7:** Associations between socio-demographic factors, consumption behaviors, and relapse in alcohol users: stages of change analysis.

Variable	Education Level	Socio-Professional Category	Readmission Within 3 Months	Family History	Comorbid Dependencies	Tobacco Dependence	Abstinence Period
*p *-value	0.110	0.341	**<0.001**	0.704	0.313	0.218	0.092
Contingency coefficient	0.334	0.282	**0.714**	0.306	0.209	0.229	0.308

Note: Bold values indicate statistically significant results (*p* < 0.05). The contingency coefficient reflects the strength of association between categorical variables.

## Data Availability

Data are available from the corresponding author upon reasonable request due to ethical reasons.

## Abbreviations

The following abbreviations are used in this manuscript:

DrInC	Drinker Inventory of Consequences—Lifetime Version
RTCQ	Readiness to Change Questionnaire—Treatment Version
DEQ	Drinking Expectancy Questionnaire
DRSEQ	Drinking Refusal Self-Efficacy Questionnaire
AUD	Alcohol use disorder
WHO	World Health Organization
QoL	Quality of life
SES	Socioeconomic status
WHOQOL-BREF	World Health Organization Quality-of-Life Scale
GDPR	General data protection regulation
FHAC	Family history of alcohol consumption
ANOVA	One-way analysis of variance
VIF	Variance inflation factor
